# Soshiho-tang water extract inhibits ovalbumin-induced airway inflammation via the regulation of heme oxygenase-1

**DOI:** 10.1186/s12906-015-0857-3

**Published:** 2015-09-18

**Authors:** Woo-Young Jeon, Hyeun-Kyoo Shin, In-Sik Shin, Sang Kyum Kim, Mee-Young Lee

**Affiliations:** K-herb Research Center, Korea Institute of Oriental Medicine, 1672 Yuseong-daero, Yuseong-gu, Daejeon, 305-811 Republic of Korea; College of Veterinary Medicine, Chonnam National University, 77 Yongbong-ro, Buk-gu, Gwangju, 500-757 Republic of Korea; College of Pharmacy, Chungnam National University, 99 Daehak-ro, Yuseong-gu, Daejeon, 305-764 Republic of Korea

**Keywords:** Soshiho-tang, Asthma, Eosinophils, Th2-type cytokines, IgE, Heme oxygenase-1

## Abstract

**Background:**

Soshiho-tang, known as Xio-hai-Hu-Tang in Chinese and Sho-Saiko-to in Japanese, has been widely used as a therapeutic agent. Its pharmacological effects include anti-inflammatory, antioxidant, antihepatic fibrosis, antitumor and immunomodulating activities. However, little is known regarding its effects on allergic asthma. Therefore, the aim of the present study was to investigate whether the Soshiho-tang water extract (SSTW) has antiasthmatic effects on airway inflammation in an ovalbumin (OVA)-induced mouse model.

**Methods:**

BALB/c mice were used as a model of asthma after induction by sensitization and challenge with OVA. We measured change in eosinophils, other inflammatory cells, and T helper 2 (Th2)-type cytokines, such as interleukin (IL)-4, IL-5, IL-13, IL-17, IL-33, and chemokine (eotaxin) in bronchoalveolar lavage fluid (BALF), presence of total and OVA-specific immunoglobulin (Ig)E in plasma, and expression of mucus production and heme oxygenase (HO)-1 protein in lung tissue.

**Results:**

Our results show that SSTW had a suppressive effect on eosinophil influx into BALF and decreased the levels of Th2-type cytokines. Moreover, SSTW exhibited a marked decrease in mucus hypersecretion, total and OVA-specific IgE levels, and significantly induced HO-1 protein expression.

**Conclusions:**

These results suggest that SSTW may be used as a valuable therapeutic agent for treating various inflammatory diseases including allergic asthma.

## Background

Asthma is a complex disorder associated with T helper 2 (Th2) immune responses to allergens characterized by airway obstruction and pulmonary inflammation that affects approximately 300 million people worldwide [1]. It is now well recognized that asthma is caused by Th2-driven inflammatory responses, which enhance airway eosinophilia and lung mucus production [2]. Allergic immune response is initiated by activation of allergen-specific Th2 cells, release of the Th2-type cytokines interleukin (IL)-4, IL-13 coupled with an increase in immunoglobulin (Ig)E, and localized eosinophilia [3].

CD4^+^ T-cells play a crucial role in immune protection via their capacity to help B cells make antibodies, recruit eosinophils to sites of inflammation, and through their production of cytokines and chemokines [4]. Th2-type cytokines, including IL-4, IL-5, IL-13, IL-17, and IL-33 produced by activated CD4^+^ T-cells, enhance IgE production, eosinophil accumulation [5], and play a central role in the pathogenesis of asthma [6]. Therefore, suppression of Th2-type cytokine production by activated CD4^+^ T-cells may prove to be a useful therapeutic approach to the treatment of inflammatory immune diseases such as allergic asthma.

A stress-response protein, heme oxygenase (HO)-1, is a cytoprotective mechanism against oxidative cellular injury [7]. It is induced by various stress stimuli, including hemin, oxidative stress, endotoxins, and hypoxia in various cell types [8]. Resent, basic study show that HO-1, a protective gene, and its overexpression have significant cytoprotection properties such as antioxidative and anti-inflammatory effects as well as mediation of cell-cycle activities [9]. Therefore, the present study focused on whether Soshiho-tang has an antiasthmatic effect via upregulation of HO-1 in an ovalbumin (OVA)-induced asthmatic model.

Soshiho-tang, known as Xio-Chai-Hu-Tang in Chinese and Sho-Saiko-to in Japanese, is frequently used for treatment of pulmonary disorders such as the common cold and pneumonitis [10]. It is composed of seven herbs: Bupleuri Radix, Scutellariae Radix, Ginseng Radix, Pinelliae Tuber, Glycyrrhizae Radix et Rhizoma, Zingiberis Rhizoma Crudus, and Zizyphi Fructus (Table 1). According to previous experimental reports, Soshiho-tang has a variety of confirmed pharmacological effects such as anti-inflammatory [11], antioxidant [12], and immunoregulation [13] activities. Several biological activities of Soshiho-tang have been reported to date; however, there are still no valid studies of its underlying OVA-induced asthmatic effects. Therefore, we investigated the antiallergic effect related to inflammation and oxidative stress of Soshiho-tang water extract (SSTW) on the treatment of bronchial asthma using an OVA-induced mouse model. To our knowledge, this is the first study to provide experimental evidence that Soshiho-tang has antiasthma effects in an OVA-induced murine asthma model.

**Table 1:** Crude components of Soshiho-tang

Scientific name	Amount (g)	Company of purchase	Source
Bupleuri Radix	11.25 (31.6 %)	Omniherb	Korea
Scutellariae Radix	7.5 (21.1 %)	HMAX	China
Ginseng Radix	3.75 (10.5 %)	Omniherb	Korea
Pinelliae Tuber	3.75 (10.5 %)	Omniherb	Korea
Glycyrrhizae Radix et Rhizoma	1.875 (5.3 %)	HMAX	China
Zingiberis Rhizoma Crudus	3.75 (10.5 %)	Omniherb	Korea
Zizyphi Fructus	3.75 (10.5 %)	Omiherb	Korea
Total amount	35.625 (100 %)		

## Methods

### Preparation of the SSTW

The water extract of Soshiho-tang (2008-KE26) was deposited in the K-herb Research Center, Korea Institute of Oriental Medicine (KIOM). Soshiho-tang was prepared in our laboratory from a mixture of chopped crude herbs purchased from Omniherb (Korea) and HMAX (China). The identity of each crude herb was confirmed by Professor Je-Hyun Lee at the Oriental College of Dongguk University (Gyeongju, Republic of Korea). A herbal decoction of Soshiho-tang was prepared with a mixture of herbal medicines according to composition in the laboratory (Table 1). The aqueous preparation were extracted in distilled water at 100 °C for 120 min. The extract solution was evaporated to dryness and then frozen to dry powder (yield, 22.9 %). The extracted Soshiho-tang powder was stored at 4 °C.

### Animals

Specific pathogen-free female BALB/c mice (seven weeks old) were purchased from the Orient Co. (Seoul, Korea). Mice were maintained in an animal facility under standard laboratory conditions for 1 week of quarantine and acclimatization prior to experiments, and provided water and standard chow *ad libitum*. The animal experimental procedures were approved by the Korea Institute of Oriental Medicine Institutional Animal Care and Use Committee and performed in compliance with the National Institute of Health Guidelines for the care and use of laboratory animals and the Korean National Animal Welfare Law.

### Induction of allergic asthma and experimental design

To induce asthma, OVA sensitization and airway challenge were performed as described previously [14, 15]. The mice were sensitized on days 0 and 14 by intraperitoneal injection of 20 μg OVA emulsified in 2 mg aluminum hydroxide in 200 μL phosphate buffered saline (PBS) buffer (pH 7.4). On days 21, 22, and 23, the mice received an airway challenge with OVA (1 %, w/v, in PBS) for 1 h using an ultrasonic nebulizer (NE-U12; Omron Corp., Tokyo, Japan). SSTW was completely dissolved in PBS and was prepared fresh daily before each treatment. SSTW was administered by oral gavage to mice at doses of 100 and 200 mg/kg once daily from days 18 to 23. Negative and positive control mice were orally administered PBS and montelukast (Mon, 30 mg/kg in PBS), respectively. Mon is a potent, selective cysteinyl leukotriene 1 (CysLT_1_) receptor antagonist [16] and was introduced into the market after successful clinical evaluation in patients with aspirin-sensitive asthma, nocturnal exacerbation of asthma, and allergic asthma [17].

At the end of OVA challenge, bronchoalveolar lavage fluid (BALF) samples were obtained from the trachea of mice and processed. Inflammatory cells were counted as described previously [14, 15]. In brief, the mice were sacrificed by intraperitoneal injection of pentobarbital (50 mg/kg; Hanlim Pharm. Co., Seoul, Korea) 48 h after the last challenge, and a tracheostomy was performed. To collect the BALF, ice-cold PBS (0.5 mL) was infused into the lung and withdrawn through tracheal cannulation three times (total volume 1.5 mL). The total inflammatory cell numbers were assessed by counting cells in at least five squares of a hemocytometer after exclusion of dead cells by trypan blue staining. To determine differential cell counts, 100 μL of BALF was centrifuged onto slides using a Cytospin unit (Hanil Science Industrial, Seoul, Korea) (200 g, 4 °C, 10 min). The slides were dried, and the cells were fixed and stained using Diff-Quik® staining reagent (B4132-1A; IMEB Inc., Deerfield, IL), according to the manufacturer’s instructions. The supernatant obtained from BALF was stored at −70 °C for biochemical analysis. Blood samples were obtained from the mice via the inferior vena cava. Plasma was collected via centrifugation (200 g, 4 °C, 10 min) and stored at −70 °C.

### Measurement of the levels of Th2-type cytokines and chemokine in BALF

The levels of IL-4, IL-5, IL-13, IL17, IL-33 and eotaxin in BALF were measured using enzyme-linked immunosorbent assay (ELISA) kits (BioSource International, Camarillo, CA) according to the manufacturer’s protocols. The detection range of IL-13 is 3.9 pg/mL to 250 pg/mL, and IL-17 is 7.1 pg/mL to 450 pg/mL, and IL-5 is 7.8 pg/mL to 500 pg/mL. The detection ranges of IL-4 are 15.6 pg/mL to 1000 pg/mL, and eotaxin and IL-33 are 31.25 pg/mL to 2000 pg/mL.

### Measurement of total and OVA-specific IgE in plasma

The levels of total IgE and OVA-specific IgE in plasma were measured as described previously [18]. Microtiter plates were coated with anti-IgE antibodies (anti-mouse IgE; 10 g/mL; Serotec, Oxford, UK) in PBS-Tween 20, and incubated with plasma sample. The plates were then washed four times, and 200 μL of o-phenylenediamine dihydrochloride (Sigma-Aldrich, St. Louis, MO) was added to each well. The plates were incubated for 10 min in the dark and the absorbance was then measured at 450 nm.

### Lung tissue histopathological studies

For histopathological examination, the lung tissues were stained as described previously [19]. The 4 % paraformaldehyde-fixed tissues were embedded in paraffin, sectioned at 4 μm thickness, and stained with hematoxylin and eosin (H&E) stain solution (hematoxylin; Sigma MHS-16 and eosin; Sigma HT110-1-32) and periodic acid − Schiff (PAS) (IMEB Inc., San Marcos, CA) to measure inflammatory cell accumulation and mucus production, respectively. Quantitative analysis for airway inflammation and mucus secretion was evaluated using a MetaMorph Offline version 7.7.0.0 image analysis software (Molecular Devices Inc., Sunnyvale, CA, USA).

### HO-1 enzyme activity in lung tissue

HO-1 enzyme activity was assessed as described previously [19]. Briefly, lungs were homogenized on ice in one volume of 100 mmol/L phosphate buffer with 2 mmol/L MgCl_2_, and centrifuged for 15 min at 18,800 g. The supernatant was used to measure HO-1 enzyme activity. ELISA assays were performed according to the manufacturer’s instructions. The HO-1 level in lung tissue was estimated using a specific mouse HO-1 ELISA kit (DAKARA, Japan). Activity values were expressed on a per-mg-protein basis.

### Immunohistochemistry

Immunohistochemistry assay were performed as described previously [19]. Paraffin sections were deparaffinized, dehydrated, and washed in PBS with 0.3 % Triton X-100. Slides were preincubated for 10 min at room temperature with 10 % goat serum to block nonspecific staining, and then incubated with mouse anti-rabbit HO-1 primary antibody (1:200; Abcam, Cambridge, UK) overnight at 4 °C. After removal of primary antibody, slides were washed and incubated with biotinylated secondary antibody at 37 °C for 1 h and then with the avidin–biotin–peroxidase complex (Vector Laboratories, Burlingame, CA) for 1 h at room temperature. Excess secondary antibody was removed, and slides were washed with PBS and incubated with 0.05 % diaminobenzidine (1:200; Millipore, Billerica, MA) for 10 min. Sections were counterstained, rinsed in PBS to terminate the reaction, and coverslipped for microscopic examination.

### Image capture and photomicrography

Photomicrographs were taken using a Photometric Quantix digital camera running a Windows program, and montages were assembled in Adobe Photoshop 7.0. Images were cropped and corrected for brightness and contrast, but were not otherwise manipulated as described previously [18].

### Statistical analysis

Data are expressed as means ± standard error of the mean (SEM). Statistical significance was determined using analysis of variance (ANOVA) followed by multiple comparison tests with Bonferroni adjustment. Differences in *P* values < 0.05 or < 0.01 were considered statistically significant.

## Results

### Effects of SSTW on number of eosinophils and other inflammatory cells in BALF

**Fig. 1:**
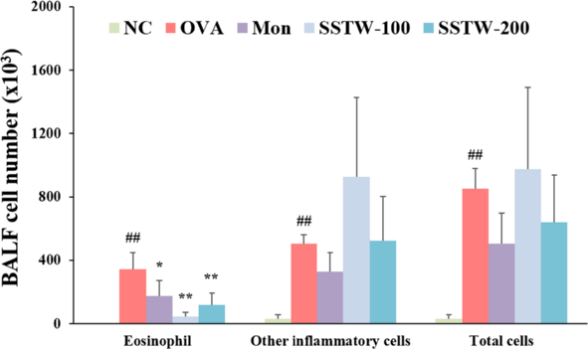
Effects of SSTW on recruitment of inflammatory cells in BALF of OVA-mice. Cells were isolated by cytospin and stained with Diff-Quick. Cell numbers were determined using a light microscope to count cells in at least five squares of a hemocytometer after excluding dead cells using trypan blue. NC, normal control group (PBS only); OVA, OVA-induced group; Mon, montelukast (30 mg/kg) + OVA-induced group; SSTW-100, SSTW (100 mg/kg) + OVA-induced group; SSTW-200, SSTW (200 mg/kg) + OVA-induced group. Each bar represents the mean ± SEM (n = 6 per group). Significant differences at ^*##*^ *P* < 0.01 and ^*#*^ *P* < 0.05 compared with the NC group. Significant differences at ^*^ *P* < 0.05 and ^**^ *P* < 0.01 compared with the OVA-induced group

To investigate the anti-inflammation effect of SSTW on the eosinophilia in OVA-induced asthmatic mice, we determined the number of total cells including eosinophils and other inflammatory cells in BALF. As shown in Fig. 1, the number of total cells, eosinophils, and other inflammatory cells in the OVA-induced group was significantly higher when compared with the NC group with PBS-induced mice. However, a positive control drug in this study, Mon group dramatically decreased the number of total cells and eosinophils compared with the OVA-induced group. Administration of SSTW (100 and 200 mg/kg) strongly exhibited a suppressive effect on eosinophilia influx into BALF.

### Effects of SSTW on OVA-induced cytokines and chemokine level in BALF

**Fig. 2:**
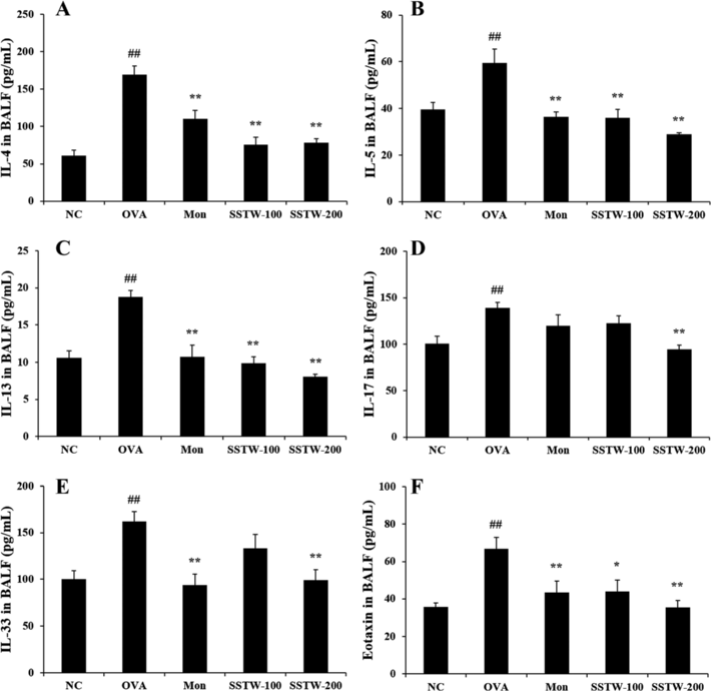
Effects of SSTW on cytokine and chemokine levels in BALF. Individual values were obtained using ELISA analysis. (**a**) IL-4 level; (**b**) IL-5 level; (**c**) IL-13 level; (**d**) IL-17 level; (**e**) IL-33 level; (**f**) eotaxin level. NC, normal control group (PBS only); OVA, OVA-induced group; Mon, montelukast (30 mg/kg) + OVA-induced group; SSTW-100, SSTW (100 mg/kg) + OVA-induced group; SSTW-200, SSTW (200 mg/kg) + OVA-induced group. Each bar represents the mean ± SEM (n = 6 per group). Significant differences at ^*##*^ *P* < 0.01 compared with the NC group. Significant differences at ^*^ *P* <0.05 and ^**^ *P* < 0.01 compared with the OVA-induced group

To determine the possible effects on the T-cell response associated with asthmatic mice, we evaluated the Th2-type cytokines and chemokine level in BALF. Figure 2 shows that the OVA-induced group had significantly elevated Th2-type cytokine and chemokine levels compared with the NC group. However, Mon- and SSTW-treated groups (100 and 200 mg/kg) had significantly decreased levels of Th2-type cytokines, including IL-4 (Fig. 2a), IL-5 (Fig. 2b), IL-13 (Fig. 2c), IL-17 (Fig. 2d), IL-33 (Fig. 2e), and chemokine such as eotaxin (Fig. 2f), compared with those from the OVA-induced group.

### Effects of SSTW on inflammatory cell accumulation and mucus production in lung tissue

To evaluate the inhibitory effect on the histological change in the lung tissue of OVA-induced asthmatic mice, we stained lung sections with H&E stain solution and PAS. The infiltration of leukocytes in the peribronchial/peribronchiolar lung tissue was not detected in the NC group with PBS-induced mice.

**Fig. 3:**
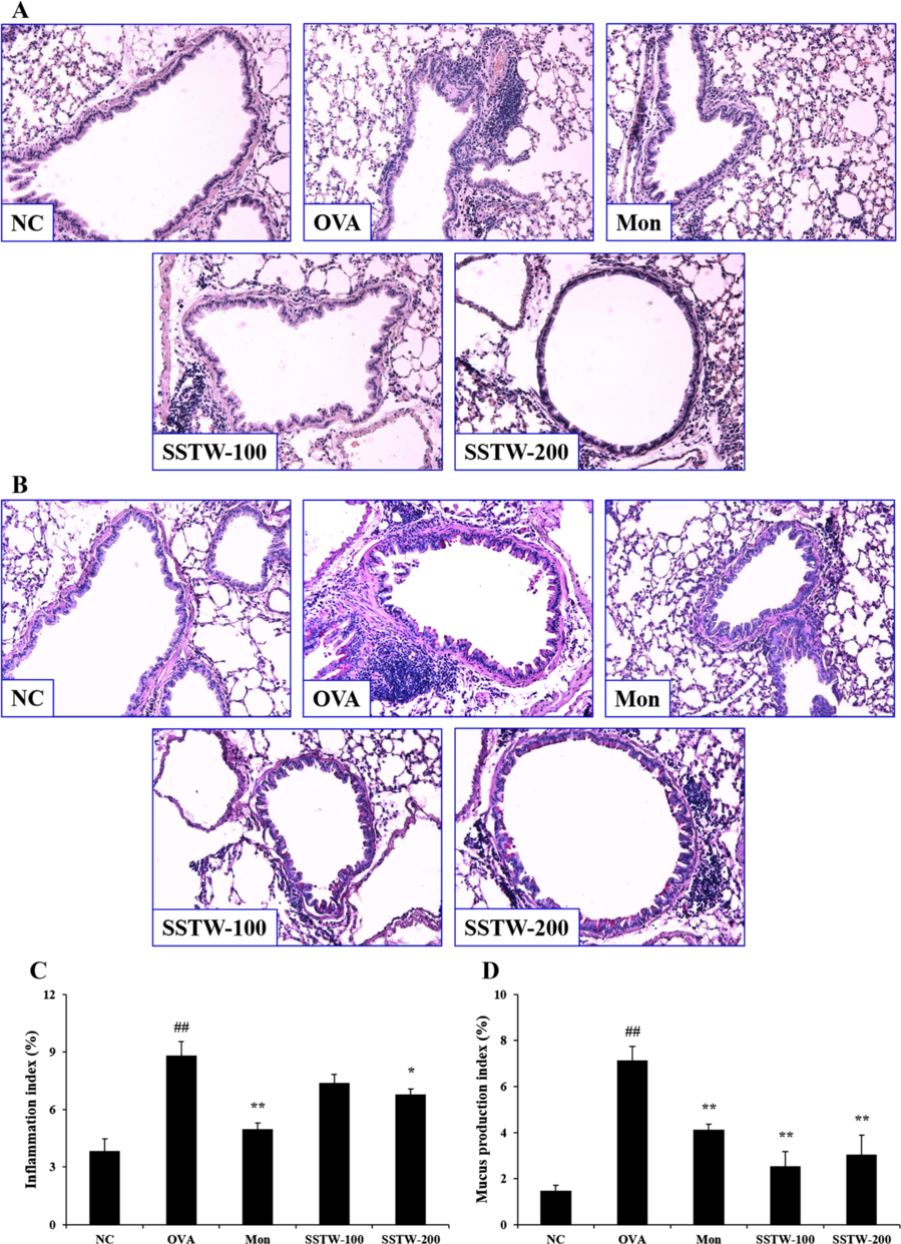
Effects of SSTW on recruitment of leukocytes and mucus production in lung tissue. Lung tissues were stained with (**a**) H&E and (**b**) periodic acid–Schiff (PAS) solution (magnification 200 ×). **a** and **b** Representative photomicrographs of lung sections are shown (n = 6 per group). **c** Inflammation index and (**d**) mucus production index were determined using an image analyzer. NC, normal control group (PBS only); OVA, OVA-induced group; Mon, montelukast (30 mg/kg) + OVA-induced group; SSTW-100, SSTW (100 mg/kg) + OVA-induced group; SSTW-200, SSTW (200 mg/kg) + OVA-induced group. Each bar represents the mean ± SEM (n = 6 per group). Significant differences at ^##^ *P* < 0.01 compared with the NC group. Significant differences at ^*^ *P* < 0.05 and ^**^ *P* < 0.01 compared with the OVA-induced group

In contrast, the OVA-induced group showed the infiltration of leukocytes such as inflammatory cell accumulation of high density. However, treatment with SSTW (100 and 200 mg/kg) effectively suppressed inflammatory cell numbers in lung tissue (Figs. 3a and c). Mucus overproduction in the OVA-induced group was clearly observed as a violet color in the bronchial airways compared with the NC group. However, administration of Mon and SSTW (100 and 200 mg/kg) significantly decreased the mucus hypersecretion in lung tissue (Figs. 3b and d).

### Effects of SSTW on the release of IgE levels

**Fig. 4:**
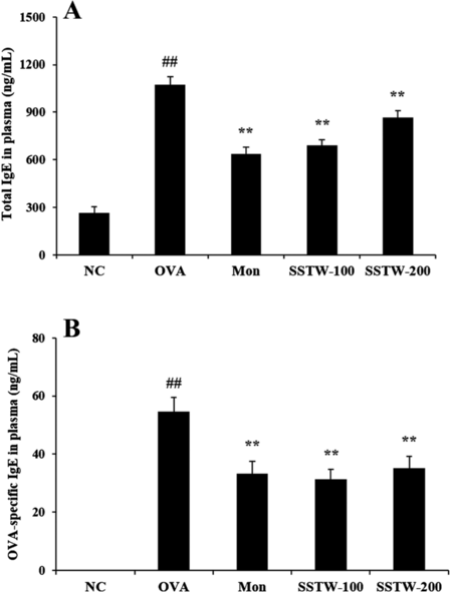
Effects of SSTW on total and OVA-specific IgE levels in plasma. Data were obtained using ELISA analysis. **a** Total IgE level and (**b**) OVA-specific IgE level. NC, normal control group (PBS only); OVA, OVA-induced group; Mon, montelukast (30 mg/kg) + OVA-induced group; SSTW-100, SSTW (100 mg/kg) + OVA-induced group; SSTW-200, SSTW (200 mg/kg) + OVA-induced group. Each bar represents the mean ± SEM (n = 6 per group). Significant differences at ^##^ *P* < 0.01 compared with the NC group. Significant differences at ^**^ *P* < 0.01 compared with the OVA-induced group

To confirm the association with Th2-type responses in the plasma of OVA-induced asthmatic mice, we measured the total and OVA-specific IgE levels in plasma. Total and OVA-specific IgE levels in plasma were markedly elevated in the OVA-induced group compared with the NC group. The Mon and SSTW groups (100 and 200 mg/kg) had significantly decreased total and OVA-specific IgE levels in plasma compared with the OVA-induced mice (Figs. 4a and b).

### Effects of SSTW on HO-1 level and protein expression

**Fig. 5:**
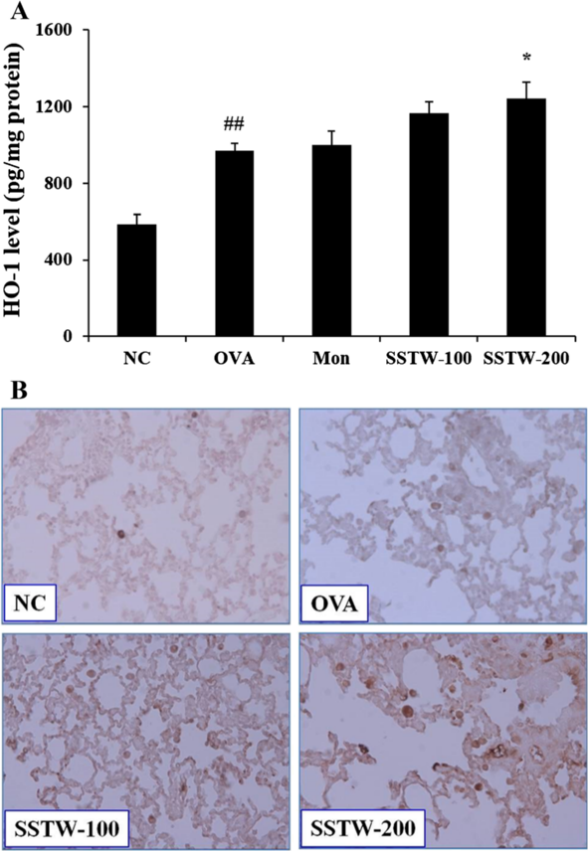
Effects of SSTW on HO-1 level and protein expression. Data were obtained using (**a**) ELISA analysis and (**b**) immunohistochemistry. NC, normal control group (PBS only); OVA, OVA-induced group; Mon, montelukast (30 mg/kg) + OVA-induced group; SSTW-100, SSTW (100 mg/kg) + OVA-induced group; SSTW-200, SSTW (200 mg/kg) + OVA-induced group. Each bar represents the mean ± SEM (n = 6 per group). Significant differences at ^##^ *P* < 0.01 compared with the NC group. Significant differences at ^*^ *P* <0.05 compared with the OVA-induced group

To determine the HO-1 protein expression associated with asthmatic mice, we evaluated the HO-1 level and protein expression by ELISA and immunohistochemistry. As shown in Fig. 5a, the HO-1 level in homogenized lung tissue significantly increased in the OVA-induced group compared with the NC group. Moreover, administration of SSTW (100 and 200 mg/kg) gradually increased more than the OVA-induced group in a dose-dependent manner. These results were consistent with immunohistochemistry analysis. HO-1 protein expression markedly increased in the OVA-induced group compared with the NC group. SSTW treatment (100 and 200 mg/kg) strongly increased HO-1 protein expression compared with the OVA-induced group (Fig. 5b).

## Discussion

The objective of this study was to evaluate the antiasthmatic effect of SSTW in an asthma-induced mouse model by OVA. We investigated the influx of inflammatory cells (particularly, eosinophils) and Th2-type cytokines including IL-4, IL-5, IL-13, IL-17, IL-33, and chemokine (eotaxin) into the BALF, IgE levels in plasma, inflammatory cell infiltration, mucus production, and HO-1 protein expression in lung tissue. Our results indicate that SSTW significantly decreased the airway inflammation by suppressing the influx of inflammatory cells as eosinophilia, increased Th2-type cytokines, chemokine, IgE levels, and mucus hypersecretion, and gradually increased the HO-1 expression related to oxidative stress.

Soshiho-tang is a traditional herbal medicine used specifically to treat alternating chills and fever, hardness and fullness in the hypochondrium, dry retching, and bitter taste in the mouth. Moreover, it is probably one of the most extensively investigated herbal formulas as experimental and clinical data have been accumulating worldwide [13]. Based on previous studies, among the constituents of the mixed herbal Soshiho-tang, we can assume that the beneficial effects in this allergic asthma model are via the antiasthmatic effect of Pinelliae Tuber [20], the antiallergic effect of Zingiberis Rhizoma Crudus [21], the anti-inflammatory effect of Scutellariae Radix [22], Ginseng Radix [23], and Zizyphi Fructus [24], the antioxidant effect of Glycyrrhizae Radix et Rhizoma [25], and the hemolytic activities of Bupleuri Radix [26]. The composition of SSTW was analyzed using high performance liquid chromatography (HPLC) in previous study [27]. The chemical standards used to identify and quantify compounds in the SSTW were as follows: liquritin, glycyrrhizin and baicalin (marker components). Previous studies demonstrated that the compounds exerts multiple biological functions and pharmacological effects such as anti-inflammatory effect of liquritin [28], and antioxidant effect of glycyrrhizin [29] and antiallergy effect of baicalin [30]. The present study aimed to investigate whether SSTW has antiallergic effects related to inflammation effects through modulation of the production of Th2-type cytokines.

The obvious mechanisms of chronic airway inflammation in asthma are incompletely known but are considered to be dependent on the sustained infiltration and activation of numerous inflammatory cells including eosinophils, neutrophils, lymphocytes, and macrophages, followed by synthesis and release of a variety of pro-inflammatory mediators and cytokines [31]. In particular, among the activated cells in the pathogenesis of allergic asthma, eosinophils are the most prominent cell type, and an increased number in the airways is correlated with the severity of asthma [32]; accordingly, selective elimination of eosinophils is often a target for the therapy of various inflammatory diseases including allergic asthma. A previous study demonstrated that recruitment of eosinophils into BALF was clearly observed as expected in the OVA-induced asthmatic mice [19]. Administration of SSTW dramatically decreased the number of eosinophils in BALF. Our results indicate that SSTW is an effective eosinophil-depleting agent. In addition, several mediators secreted from eosinophils and lymphocytes can amplify portions of the inflammatory cascade; they can increase airway hyperresponsiveness, inflammatory cell accumulation, and stimulate mucus secretion, both of which can contribute to airway obstruction and remodeling [33]. In our study, we found elevated leukocyte infiltration, goblet-cell hyperplasia, and mucus hypersecretion, confirming a previous study [19]. To support the previous results, our histopathological findings (H&E and PAS stain) demonstrate that SSTW significantly decreased the OVA-induced leukocyte infiltration, goblet-cell hyperplasia, and mucus hypersecretion.

Initiation of allergic response occurs with allergen presentation by antigen-presenting cells to CD4^+^ T cells. Antigen-activated CD4^+^ T cells induce several characteristic features of asthma, including the secretion of Th2-type cytokines such as IL-4, IL-5, and IL-13, which are responsible for IgE production by B cells and eosinophil activation and recruitment [34, 35]. IL-4 is the most important Th2-type cytokine in inducing isotype switching to IgE in B lymphocytes [36]. IL-5 plays a major role in the maturation and recruitment of eosinophils into the airway [37]. IL-13 induces many features of allergic lung disease, including goblet cell metaplasia and mucus hypersecretion, which all contribute to airway obstruction [38]. IL-17 induces the production of many other cytokines (such as IL-6, G-CSF, GM-CSF, IL-1β, TGF-β, and TNF-α), chemokines, and prostaglandins (e.g., PGE2) from many cell types such as fibroblasts, endothelial cells, epithelial cells, keratinocytes, and macrophages [39]. IL-33 is a potent inducer of proinflammatory cytokine and chemokine production by mast cells. It also induces the degranulation of IgE-primed mast cells and enhances mast-cell maturation and survival [40]. Eotaxin plays a role in airway remodeling through recruitment of eosinophils and mast cells, which have profibrogenic activity [41]. Thus, these Th2-type cytokines induce inflammatory responses, such as inflammatory cells infiltration, eosinophil activation, IgE production, and mucus secretion [42]. Many studies suggest that the anti-inflammatory effects of Th2-type cytokines occur via diminishing its production in a mouse model of allergic asthma [43, 44]. Our findings indicate that SSTW significantly decreased Th2-type cytokine production. This anti-inflammatory effect of SSTW may be responsible, at least in part, for the decreased recruitment of eosinophils into the lung. IgE may be an important target in treatments for allergy and asthma and is closely associated with Th2-type responses. Administration of SSTW markedly attenuated the increased total and OVA-specific IgE levels in plasma. These findings support the evidence that SSTW regulates the Th2-type immune response in an asthmatic mouse model. Therefore, SSTW may have potential as an effective antiasthma agent through decrease of IgE levels.

An imbalance between the oxidative forces and the antioxidant defense systems favoring an oxidative injury has been implicated in the pathogenesis of asthma [45]. Oxidative injury leads to an augmentation of the existing inflammation that is a hallmark of asthma. One of these defense mechanisms is the induction of a stress-response protein, HO-1. Therefore, to determine a factor related to oxidative stress we measured HO-1 expression. HO-1, a protective gene, is a highly inducible enzyme that defends cells against oxidative stress and is induced in a mouse model of asthma. Moreover, its deficiency leads to an increase in chronic inflammation and leukocyte recruitment [46]. Previous studies reveal that the induction of HO-1 helps to reduce tissue injury and inflammation in a models of experimental asthma [47–49]. It was suggested that HO-1 induction occurs as a protective mechanism against oxidant-mediated cellular injury representing reactive oxygen species production [50]. Our results confirm that SSTW overexpressed OVA-induced production of HO-1, suggesting that the protective effect of SSTW is related to its reduction of oxidative stress. Increased HO-1 protein expression may be due to the induction of the enzyme by inflammatory mediators present in the asthmatic airway. Many cytokines and oxidants involved in asthmatic inflammation including interleukins are able to induce HO-1 expression in cell lines and tissues under experimental conditions [51]. Taken together, our findings demonstrate that SSTW provides cytoprotection through overexpression of HO-1 induced by Th2-type cytokines.

Mon is a CysLT_1_ receptor antagonist used for the maintenance treatment of asthma to relieve allergic symptoms. Mon inhibits the action of leukotriene D4 on the CysLT_1_ receptor in the lung, which reduces bronchoconstriction and airway inflammation [52, 53]. In present study, Mon reduces the eosinophilic airway inflammation, and decrease the release of Th2-type cytokines, chemokine, IgE and mucus hypersecretion. However, the regulation of HO-1 have not worked. Treatment of SSTW reduces asthmatic responses by providing similar result to Mon. In addition, activation of HO-1 attenuated airway inflammation by administration of SSTW in mice with OVA-induced asthma. Considering the overall results, our findings demonstrate that SSTW exerts antiasthmatic effects on OVA-induced airway inflammation, and that HO-1 induction may be at least partly responsible for its action.

## Conclusions

Our findings demonstrate that SSTW significantly inhibits OVA-induced asthmatic reaction by reduction of leukocytes, eosinophilic inflammation and downregulation of Th2-type cytokines, chemokines, and decrease of mucus hypersecretion, IgE levels and upregulation of HO-1 expression. Therefore, SSTW may prove to be a useful therapeutic agent for the treatment of allergic airway diseases.

## Abbreviations

ANOVA: Analysis of variance
BALF: Bronchoalveolar lavage fluid
CysLT_1_: Cysteinyl leukotriene 1
ELISA: Enzyme-linked immunosorbent assay
HO: Heme oxygenase
H&E: Hematoxylin and eosin
Ig: Immunoglobulin
IL: Interleukin
Mon: Montelukast
OVA: Ovalbumin
PAS: Periodic acid − Schiff
SEM: Standard error of the mean
SSTW: Soshiho-tang water extract
Th2: T helper 2
